# Innovative and Promising Strategies to Enhance Effectiveness of Immunotherapy for CNS Tumors: Where Are We?

**DOI:** 10.3389/fimmu.2021.634031

**Published:** 2021-06-07

**Authors:** Concetta Quintarelli, Antonio Camera, Roselia Ciccone, Iside Alessi, Francesca Del Bufalo, Andrea Carai, Giada Del Baldo, Angela Mastronuzzi, Biagio De Angelis

**Affiliations:** ^1^ Department Onco-Hematology, Cell and Gene Therapy, IRCCS, Bambino Gesù Children’s Hospital, Rome, Italy; ^2^ Department of Clinical Medicine and Surgery, University of Naples Federico II, Naples, Italy; ^3^ Neurosurgery Unit, Department of Neurological and Psychiatric Sciences, IRCCS Bambino Gesù Children’s Hospital, Rome, Italy

**Keywords:** CNS, brain tumor, immunotherapy, chimeric antigen receptor, T cell receptor, oncolytic virotherapy, vaccination, immunocheck point inhibitors

## Abstract

Although there are several immunotherapy approaches for the treatment of Central Nervous System (CNS) tumors under evaluation, currently none of these approaches have received approval from the regulatory agencies. CNS tumors, especially glioblastomas, are tumors characterized by highly immunosuppressive tumor microenvironment, limiting the possibility of effectively eliciting an immune response. Moreover, the peculiar anatomic location of these tumors poses relevant challenges in terms of safety, since uncontrolled hyper inflammation could lead to cerebral edema and cranial hypertension. The most promising strategies of immunotherapy in neuro-oncology consist of the use of autologous T cells redirected against tumor cells through chimeric antigen receptor (CAR) constructs or genetically modified T-cell receptors. Trials based on native or genetically engineered oncolytic viruses and on vaccination with tumor-associated antigen peptides are also under evaluation. Despite some sporadic complete remissions achieved in clinical trials, the outcome of patients with CNS tumors treated with different immunotherapeutic approaches remains poor. Based on the lessons learned from these unsatisfactory experiences, novel immune-therapy approaches aimed at overcoming the profound immunosuppressive microenvironment of these diseases are bringing new hope to reach the cure for CNS tumors.

## Introduction

Brain tumors are the most common solid tumors of children (1, 2). Standard therapy for these diseases includes surgical resection, radiation, and, in selected cases, chemotherapy. Despite aggressive treatment, many patients have a poor long-term outcome or, frequently, develop treatment-related long-term sequelae, including hormone dysfunction, sensory-motor and neurocognitive impairments (3). In the last decades, information on molecular pathways responsible for the development of these tumors has increased as result of extensive knowledge on the genomic, epigenetic, and transcriptomic landscape. This allows for a better tumor classification and provides valuable information for target therapies but does not significantly improve survival for the most aggressive brain tumors. Immunotherapy represents an innovative and attractive approach potentially able to eradicate cancer cells, sparing adjacent normal brain tissue. This peculiarity is relevant for CNS tumors, which cannot be surgically resected due to their location in the brainstem. However, several factors may hinder the efficacy of cell immune therapy in the contest of CNS tumors. Indeed, the brain was historically considered an immune-privileged site due especially to the presence of several physical barriers, including blood–brain barrier (BBB), blood-meningeal barrier, and choroid plexus barrier (also known as blood–cerebrospinal fluid (CSF) barrier) (4, 5). Moreover, the adaptive response to CNS antigens is strongly reduced due to lack of (efficient) lymphatic drainage (6). A recent report describes the presence of lymphatic system in the meninges, allowing T cells to reach the draining cervical lymph nodes for antigen presentation (7). Physiologically, although resting T cells do not cross the BBB, they traffic from meningeal blood vessels into the CSF, where cells can enter the brain parenchyma *via* the pia mater or choroid plexus. By contrast, activated T cells are able to traverse the BBB trough the capillary tight junctions. For this reason, immunotherapy approaches associated with *in vivo* or *ex vivo* activation of T cells may potentially provide a potent tool to facilitate the penetration of the immune system toward the BBB.

Moreover, the development of an effective immunotherapy approach should take into consideration that normal brain parenchyma has evolved to protect itself against an immunologic attack (8), characterized by the paucity of professional antigen-presenting cells (APC) (9) and by the downregulated expression of the major histocompatibility complex (MHC), both features limiting antigen presentation (10).

New evidence shows that the tumor microenvironment (TME) plays a key role in negative modulation of immunotherapeutic approaches.

Glioblastoma (GBM) is one of the best characterized CNS tumors with regard to TME. It is characterised by abundance of cytokines [in particular interleukin-6, interleukin-10, transforming growth factor-beta (TGF-β) and prostaglandin-E] secreted by tumor cells, microglia and/or tumor-associated macrophages (TAMs), exerting strong immunosuppressive activity toward the inhibition of Natural Killer (NK)-cells and T-cells, as well as enhancing T-cell apoptosis, skewing TAM phenotype, and downregulating MHC expression on both tumor cells and APC (11, 12). Moreover, TAM are the most abundant stromal cell type in large number of cancers, including GBM (13) and brain metastases (14), and can represent up to 30–50% of the tumor mass. Indeed, the presence of TAMs, often associated with poor patient prognosis (15), fosters tumor growth, controls metastasis and affects therapeutic response (16, 17).

TME hypoxia is another relevant regulator of immunosuppressive pathways. In particular, TME milieu regulates STAT3 activation associated to hypoxia-inducible factor-1 alpha induction that in turn increase vascular endothelial growth factor (VEGF) expression. This signaling cascade leads to activation of T regulatory cells, and inhibition of APC. For this reason, targeted delivery of cytokines/chemokines has been considered to reduce the negative impact of the TME in the immune-system control of CNS tumors (18–20).

CNS inflammation is another relevant factor to be considered in the context of immunotherapy for CNS tumors. Recent insights into neuro-inflammation indicate that immunocells represent a cell component critical in regulating CNS inflammation. In particular, IL1β, besides its function as key element in inflammation, has a pivotal role in inducing a slow glioma-initiating cell (GIC) transition into a mesenchymal (MES) state (reactive-astrocyte-like cell state), influencing the tumor response to therapy, as well as the development of resistant tumor clones with a peculiar DNA methylation profile (21).

Another relevant factor to be considered in developing an effective immunotherapy for CNS tumor is the strong exhaustion profile induced *via* continuous TCR/CAR signaling and observed in tumor-infiltrating lymphocytes (TIL) from patients with brain neoplasia, associated to a significant reduction of IL2, IFN-γ and TNFα production (22, 23). In GBM patients, PD-1, LAG-3, TIGIT, and CD39 were found highly expressed on CD8+ TILs (23), supporting the reason to deeply investigate the interplay between PD-1 receptor and its ligand PD-L1 in CNS tumors. In particular, PDL-1 was not only described to be expressed on GBM cells, promoting from one end T cell inhibition and to the other end the invasion of tumor cells in the brain tissue (24, 25) but also on TAMs (26), heightening their immunosuppressive role in the brain TME (27).

These evidences led to the clinical exploration of the use of monoclonal antagonist antibodies directed towards PD1 and PD-L1, with clinical trials enrolling patients with recurrent GBM (24) (**Table 1**).

**Table 1:** Summary of recruiting or completed clinical trials covering brain tumor immuno-therapeutic approaches.

Treatment	Strategy	Brain tumor	Routes of administration	Phase	Clinical Trial Number (recruiting or completed) or PMID
Autologous NK cells administered in combination with IFN-β	Un-modified Adoptive Cell	Malignant gliomas	focal and intravenous injections	Phase I	PMID: 15274367
Autologous tumor infiltrating lymphocytes (TILs) and recombinant interleukin-2 (rIL-2)	Un-modified Adoptive Cell	Malignant gliomas	intratumoral infusions	Phase I	PMID: 10778730
Autologous lymphokine-activated killer (LAK)	Un-modified Adoptive Cell	GBM	Intralesional LAK Cell Therapy	Phase I/II	NCT 00331526
Autologous Lymphoid Effector Cells ((Cytotoxic T cells and Natural Killer)) Specific Against Tumor-cells (ALECSAT)	Un-modified Adoptive Cell	GBM	intravenous infusios	Phase I	NCT01588769
Autologous CMV-specific T-cell therapy and chemotherapy	Virus specific Adoptive Cell	Recurrent GBM	intravenous infusion	Phase I	ACTRN12609000338268
Autologous polyclonal CMV pp65-specific T cells	Virus specific Adoptive Cell	GBM	intravenous infusion	Phase I/II	PMID: 32299815
Neoadjuvant Nivolumab treatment	Immunomodulation	GBM	intravenous infusion	Phase II	NCT02550249
Nivolumab plus ipilimumab treatment	Immunomodulation	Brain metastases of Melanoma	intravenous infusion	Phase II	NCT02320058
Nivolumab *vs* Temozolomide (TMZ) Each in Combination With Radiation Therapy	Immunomodulation	Unmethylated MGMT (Tumor O-6- methylguanine DNA Methyltransferase) Glioblastoma	intravenous infusion and oral administration	Phase III	NCT02617589
HSV-1 G207 treatment	Oncolytic virus (OV) Approaches	GBM and AA	Intralesional Therapy	Phase I	PMID:10845725; PMID:18957964
HSV-1 G207 treatment combined with a single 5 Gy dose of focal radiation within 24 h	Oncolytic virus (OV) Approaches	Progressive or Recurrent Malignant Supratentorial Brain Tumors	intratumoral inoculation	Phase I	PMID: 28319448
Rindopepimut with temozolomide treatment	Vaccination	EGFRvIII-expressing glioblastoma	Rindopepimut: monthly intradermal injection Temozolomide: oral administration	Phase III	PMID: 28844499
Heat-Shock Protein Peptide Complex-96 (HSPPC-96) vaccine	Vaccination	GBM	intradermal injection	Phase II	NCT00293423.
autologous formalin-fixed tumor vaccine (AFTV)	Vaccination	GBM	intradermal injection	Phase I/IIa	UMIN000001426
HLA-A24–restricted candidate peptides (ITK-1) vaccine	Vaccination	GBM	intradermal injection	Phase I	PMID: 21149665

**Figure 1:**
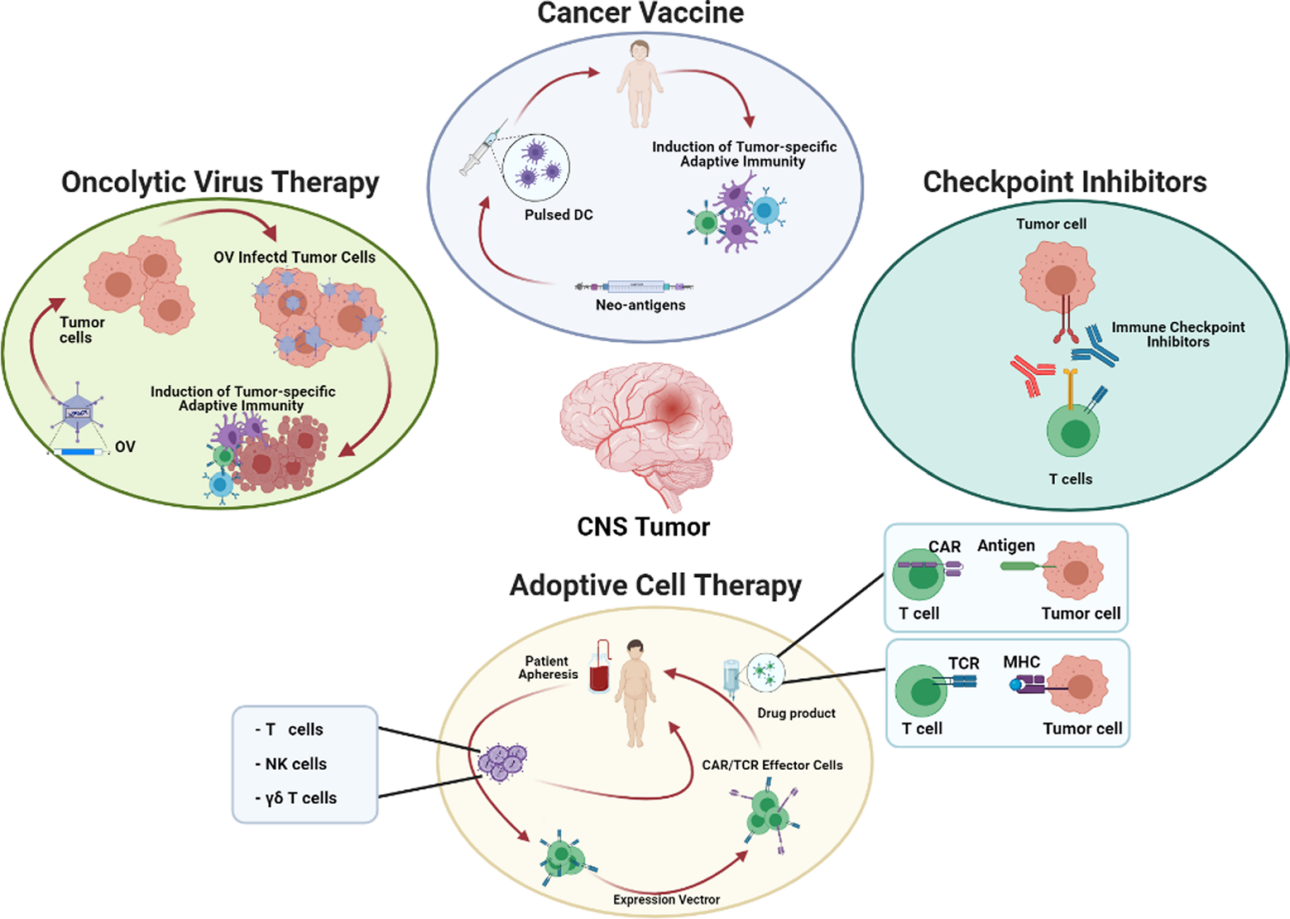
Potential strategies for the treatment of brain tumors. The cartoon summarizes the main immunotherapeutic approaches currently used in clinical trials, subdivided in four major categories: 1) Cancer Vaccine based on Dendritic Cells (DC) pulsed with neoantigens or electropored/infected to transfer neo-antigen genes; 2) Checkpoint Inhibitors based on the use of antibody blocking relevant exhaustion pathway in T cells; 3) Adoptive Cell Therapy based on genetic modification of effector cells with expression vector for TCR or CAR; 4) Oncolytic Virus Therapy based on tumor cell infection, and the consequent activation of adaptive immunity. DC, Dendritic Cells; CAR, Chimeric Antigen Receptor; TCR, T Cell Receptor; MHC, Major histocompatibility complex; NK, Natural Killer cells; OV, Oncolytic virus.

Finally, encouraging results were observed in CNS neoplasia treated with Adoptive Cell Transfer (ACT), of both un-modified or gene-modified immune cells, as well as tumor associated vaccines and oncolytic virus (**Figure 1**).

In the present review, we analyze several aspects that limit efficacy of adoptive cell therapy in patients with CNS tumors and present strategies to overcome these hurdles.

## Non-Engineered Immune Cell Approaches

Several groups proved that CNS tumors can be targeted by conventional α/β T, NK and γ/δ T cells, although their activity is significantly modulated by the TME.

NK cells play a relevant role in immunity against tumors, without requiring antigen priming or HLA restriction for displaying their activity (28). These advantages are particularly relevant for CNS cancers where the antigenic landscape is extremely heterogeneous, limiting the application of adoptive T-cells. As previously mentioned, CNS tumor cells often downregulate self-markers like HLA class I, allowing them to evade T-cell mediated lysis; however, this renders them potentially more prone to lysis by NK cells. GBM cells do express several activator ligand for NK receptors, as for example MHC class I polypeptide-related sequence A (MICA) (29) ligand for NKG2D^+^ present on NK cells, as well as CD155 and CD112 recognized by TIGIT and CD96 receptors (30). Indeed, it has been proved that NK cells exert lytic activity toward Medulloblastoma (MB) and GBM cell lines, both in *in vitro* (31–33) and *in vivo* models (31, 34, 35). Nevertheless, NK cell activity needs to be optimized in order to overcome inhibition exerted by tumor intrinsic and TME-associated factors. In particular, a pivotal trial (**Table 1**) has been conducted to test safety and efficacy of autologous NK cells infused in combination with IFN-β to improve NK-cell-mediated cytotoxicity (36). Four weeks after infusion, this led to either stable disease or a measurable response without severe toxicity. These results are encouraging, although no complete response was achieved. For this reason, new strategies were considered, both in the pre-clinical and the clinical setting, to improve NK-cell mediated antitumor activity (31, 37), including their genetic modification by CAR (38, 39). Unlike NK cells, cell therapies with autologous TILs are HLA-restricted, and are based on the tumor recognition toward the T-cell receptor (TCR). TILs specific for somatic mutations have demonstrated significant clinical responses in both primary and metastatic cancers (40–42). Indeed, TILs, locally infused through an Ommaya reservoir and combined with IL2, has been investigated in glioma patients (43), resulting in no significant (Grade 3 or 4) complications, the best response being represented by partial response in three out of six patients.

Although these studies provided evidence that non-engineered immune cells may traffic to CNS and exert measurable antitumor activity, technical issues, mainly associated to difficulties to isolate and expand TILs from tiny fractions of primary brain tumors, have hampered the wide clinical application for this approach in CNS tumors.

Some groups reported that antigens and DNA from human cytomegalovirus (CMV) can be detected in GBM tissues but not in surrounding healthy tissue (44–46). This observation provided the rationale for hypothesizing alternative virus-specific ACT approaches for CNS tumors. However, the clinical relevance of this evidence is still controversial (47, 48). Indeed, it was also proved that the frequency of pp65-specific T cells in CMV-seropositive GBM patients was decreased in comparison to healthy donors (49). Although CMV is not classified as an oncogenic virus, its role in modulation of cell proliferation, angiogenesis, and immune evasion is well known (50). In the context of brain neoplasia, human CMV increases glioblastoma neurosphere proliferation and p-STAT3 levels, these findings suggested the existence of an association between CMV infection and STAT3-dependend modulation in glioma formation/progression, together with tumor suppressor mutations (51). Consistent with these findings, CMV seropositivity correlates with worse prognosis in GBM patients (52). This discovery provides the basis of viral antigen targeting immune therapies in GBM. In this context, besides the incidental observation in one patient of a robust CMV pp65-specific CD8+ cell response after injection of autologous dendritic cells pulsed with an autologous tumor lysate (53), the use of CMV-specific T-cell therapy did not show a significant efficacy in a Phase I pivotal study for GBM patients (54), or in a larger cohort of patients treated at MD Anderson Cancer Center (55).

These preliminary clinical data support the hypothesis that the development of effective immunotherapeutic strategies for CNS tumors requires a robust understanding of factors regulating the activity of the effector cells in the CNS tumor lesions. Several groups proved that un-modified immune cells therapy is significantly potentiated when administered in concomitance to hematopoietic stem and progenitor cells (HPSC). Mitchel’s group (56, 57) elegantly proved, in MB and GBM animal models, that transfer of HSPCs (in particular, CCR2+ HSCs) (57) with concomitant cell therapy approach led to the production of activated Dendric Cells (DCs) (CD86+CD11c+MHC-II+ cells). These intratumoral DCs largely replaced abundant host myeloid-derived suppressor cells. This mechanism was strictly related to the T-cell-released IFNγ that leads to the differentiation of HSPCs into DCs, activation of T cells and rejection of intracranial tumors (56). In line with these findings, it should also be mentioned that macromoleculs such cytokines, can reprogram the TME, but systemically delivered cytokines have limited access to the CNS.

## Engineering T Cells To Improve Adoptive Cell Therapy in CNS Neoplasia

RNA-modified T cells have been considered in order to deliver cytokines directly into brain tumors to bypass the hurdles to access the CNS. In a GBM murine model, T cells modified with GM-CSF RNA delivered this cytokine into brain tumors, significantly prolonging overall survival of the animals (18). In particular, GM-CSF recruited DCs in to the tumors, induced their differentiation and maturation, leading to activation of TILs (58).

### T Cell Receptor (TCR) Redirected T Cells to Target CNS Tumor Cells

TCR-based adoptive T-cell therapies allow the genetic redirection of T cells on specific tumor targets in a HLA-restricted fashion (59). TCR-based ACT has produced preliminary promising clinical results in extracranial solid tumor (60, 61) and in acute leukemia (62), whereas in brain tumors, the clinical field is still largely unexplored. At the preclinical level, several tumor antigens have been evaluated for TCR approaches in CNS neoplasms, also considering the evidence that tumor-associated antigen (TAA)-reactive T-cells could be detected in patients with glioma (63), suggesting that in this type of cancer the intratumor DCs can process TAA, leading to the emergence of tumor-specific T cells. Between the different categories of TAAs (64), cancer/testis antigens (CTA) are overexpressed in most human cancers due to promoter demethylation in tumor elements (65, 66), whereas their expression in healthy tissues is restricted to germ cells that, lacking the major histocompatibility complex (MHC), are not targeted by T cells.

Among the members of the CTA family, PRAME (antigen preferentially expressed in melanoma) is expressed in leukaemia (67), some solid tumors (68), with a restricted expression in healthy tissues (69). In brain tumors, the CTA PRAME is detectable in about 80% of MB tissues, independently from the molecular and histopathologic subgroups (70–72), with its high expression being correlated with patient dismal overall survival (72). We recently showed that PRAME-TCR T cells are capable of killing MB cells, *in vitro* as well as in murine xenograft *in vivo* models (72), representing an innovative, effective strategy for patients with MB, in the absence of significant toxicity.

NY-ESO-1 (New York oesophageal squamous cell carcinoma 1) is a CTA expressed in ovarian cancer (22.6%), oesophageal cancer (25.3%) and lung cancer (28.6%) (73). Discordant data were reported for NY-ESO-1 expression in glioma; most cases, were present at a very low level (63, 74), while the DNA‐demethylating agent, as 5‐aza‐2′‐deoxycytidine, markedly reactivated its expression in GBM but not in normal human cells (74). For this reason, demethylating agents have been administered in combination with ACT approaches targeting NY-ESO-1 (75). These preclinical studies led to a clinical investigation of the administration of T cells expanded *ex vivo* through the stimulation with APCs (represented by CD4+ T cells exposed to DNA-demethylating agent to induce the expression of high level of CTA). Among the 25 GBM patients enrolled in the Phase I clinical trial (NCT01588769), tumor regression was observed in three patients (76).

Another category of relevant TAA is represented by neoantigens generated by genetic mutation in cancer cells. One example is represented by H3.3 K27M (lysine 27 to methionine substitution in histone H3) mutation, present in 70% of DIPG patients and associated to poor overall survival (77, 78). HLA-A*02-restricted epitope from H3.3 K27M mutated protein was used to generate tumor specific T cell clone with high affinity for the selected peptide. From this T cell clone, TCR α- and β-chains were sequenced to generate H3.3K27M specific TCR, that transferred in polyclonal T cells was able to specifically redirect effector gene modified T cells toward H3.3K27M+ glioma cells *in vitro*, but also when adoptively transferred into intracranial glioma xenografts mice (79).

Tumor-specific-TCR engineered T cells represent an interesting approach, allowing to target proteins with a wide and specific expression in tumor cells even when their location is subcellular. This feature enables the ability to largely broaden the range of the targetable antigens, in regard to those suitable to be recognized by standard antibody-based immune-strategy. High affinity/avidity TCRs are described to recognize target cells even at low epitope densities (80). Moreover, considering the high cellular and molecular heterogeneity in CNS tumors (81), we could also take into consideration the development of TCRs targeting multiple antigens to avoid the rapid subclone tumor selection due to the targeting of a single peptide. Regardless of these great features and versatility, the efficacy of TCR T-cells is strictly related to the HLA-peptide presentation, which is the major reason for treatment failure in those tumors characterized by HLA down-regulation (82, 83). To overcome this issue, we have recently showed that IFN-γ treatment can re-establish HLA expression on brain tumor cells (72).

### CAR T-Cell Approaches in CNS Tumors

T cells genetically modified with CAR emerged in recent years as a promising immunotherapeutic approach, which could mediate a non-MHC-restricted anti-tumor response (84). CARs are artificial receptors composed of a region targeting a specific antigen linked, through an intracytoplasmic domain, to the T-cell activation domain CD3zeta chain (first-generation CAR). The target specific region can comprise a single-chain variable fragment (scFv) obtained from a specific monoclonal antibody, as well as a receptor domain binding a specific ligand expressed on tumor cells.

The intracytoplasmic domain can be enriched by either one or two costimulatory molecules (i.e. CD28, OX40, 4-1BB, or other) to generate second- or third-generation CARs, respectively (85). Clinical trials based on autologous CAR T-cells achieved unprecedented results for the treatment of haematological neoplasia. Two CD19-specific CAR T-cell products, namely Kymriah^®^ (Novartis) and Yescarta^®^ (Kite Pharma), have been recently approved by the US Regulatory Agency Food and Drug Administration and subsequently by the European Medicines Agency for treating B cell ALL and diffuse large B-cell lymphoma (DLBCL), respectively (86). The therapeutic efficacy of CAR T-cells beyond hematopoietic cancers is less clear to date, there is limited documented information on antitumor activity against solid neoplasms. Several reports on CAR.CD19 T cells have proven that the genetically modified cells can cross the BBB (87, 88). A patient with primary refractory DLBCL involving the brain parenchyma who achieved complete remission after CAR.CD19 T-cell infusion, in the absence of cytokine release syndrome or neurotoxic effects, provides strong support that CAR T cells have the capacity to penetrate the CNS (89). Moreover, in the context of CNS, intrathecal and intraventricular administration of CAR T-cells was tested in patients with GBM. The treatment was well tolerated, without cytokine release syndrome or severe neurotoxicity (90–92).

Different approaches with CAR T-cells have been evaluated for the treatment of patients with GBM and/or MB, also in phase I/II clinical trials, by targeting one or more of the following targets (**Table 2**):

**Table 2:** Summary of recruiting or completed clinical trials related to the use of CAR-T cells in the treatment of brain tumors.

Treatment	Target	Strategy	Costimulatory domains	Brain tumor	Routes of administration	Phase	Clinical Trial Number (recruiting or completed)
h IL13(E13Y)-zetakine CD8+ CTL	IL-13Rα2	I CAR T	none	Recurrent or Refractory High-Grade Malignant Glioma	intravenous infusion	Phase I	NCT00730613
IL13Ralpha2-specific Hinge- optimized 41BB-co- stimulatory CAR Truncated CD19-expressing T Cells		II CAR T	4-1BB	Recurrent or Refractory Glioblastoma	*CAR T Cells*: via intratumoral, intracavitary, or intraventricular catheter	Phase I	NCT02208362
IL13Rα2-Targeted CAR T Cells Combined With Checkpoint Inhibitor		II CAR T± Nivolumab and Ipilumab	4-1BB	Recurrent or Refractory Glioblastoma	*Nivolumab and Ipilumab*: intravenous infusion *CAR T Cells*: via Rickham catheter (ICV/intracranial ICT)	Phase I	NCT04003649
CAR T-EGFRvIII T cells	EGFRvIII	II CAR T	4-1BB	Patients With Residual or Reccurent EGFRvIII+ Glioma	intravenous infusion	Phase I	NCT02209376
CAR T-EGFRvIII T cells + Aldesleukin		III CAR T	CD28.4-1BB	Malignant Gliomas Expressing EGFRvIII	intravenous infusion	Phase I/II	NCT01454596
EGFR806-specific CAR T		II CAR T	4-1BB	EGFR-positive Recurrent or Refractory Pediatric CNS Tumors	Locoregional administration	Phase I	NCT03638167
CMV-specific Cytotoxic T Lymphocytes Expressing CAR-HER2	HER2	II CAR T	CD28	glioblastoma multiforme (GBM).	intravenous infusion	Phase I	NCT01109095
Memory-enriched autologous HER2(EQ)BBzeta/CD19t+ T cells		II CAR T	4-1BB	Recurrent Brain or Leptomeningeal Metastases	intraventricular administration	Phase I	NCT03696030
HER2-CAR T cell		CAR T	not specified	CNS Tumors	ARM A: Tumor Cavity Infusion ARM B:Ventricular System Infusion	Phase I	NCT03500991
HER2-specific CAR T cell		CAR T	not specified	CNS Tumors	Intracranial Injection	Phase I	NCT02442297
(C7R)-GD2.CART cells	GD2	CAR T	not specified	high grade glioma (HGG) or diffuse intrinsic pontine glioma (DIPG)	intravenous infusion	Phase I	NCT04099797
14g2a-CD8.BB.z.iCasp9		CAR T	4-1BB	Diffuse Intrinsic Pontine Gliomas (DIPG) and Spinal Diffuse Midline Glioma (DMG)	intravenous infusion	Phase I	NCT04196413
B7H3-CAR T cells	B7-H3	CAR T	not specified	Diffuse Intrinsic Pontine Glioma/Diffuse Midline Glioma and Recurrent or Refractory Pediatric Central Nervous System Tumors	via an indwelling catheter into the tumor resection cavity or ventricular system	Phase I	NCT04185038

***Interleukin-13 receptor alpha2 (IL-13Rα2)*** is over-expressed in up to 75% of the patients with GBM but not in normal brain (2, 93, 94). Both first and second-generation CARs, have been successfully used to target IL-13Rα2 (90, 92). This CAR recognizes with high affinity IL13Rα2 *via* a membrane-tethered IL-13 ligand and starts cytolytic killing (95). Moreover, targeting of IL-13Rα2 positive tumors by CAR T cell approach has been also explored in Phase I clinical trial (90, 92), proving the safety and feasibility of intracranial administration of IL-13Rα2-CAR T cells, administered toward multiple routes of infusion. These trials, pioneers of the field, were extremely relevant since underlying that in the majority of the cases, a patient’s response (whereas partial and transient) was observed in the absence of serious adverse events (90). In the path to optimize the approach and reach more impact on patient outcome, T cell population enriched in central memory cells (91) were considered during the manufacturing process as well as the CAR design was optimized by incorporating 4-1BB (CD137) as a costimulatory domain (96), to achieve a prolonged *in vivo* survival.

Finally, an innovative and intriguing approach to enhance CAR. IL-13Rα2 T cell antitumor activity toward self-renewing GBM stem cells (GSCs), has been recently discovered by the application of whole-genome clustered randomly interspersed short palindromic repeats (CRISPR)-knockout screen applied to both CAR T cells and GSCs (97). In particular, TLE4 or IKZF2 gene targeting were associated to an increased and long-term efficacy of CAR T cells in this model, with a significant reduction in exhaustion signal of the T cells, whereas knockout of RELA or NPLOC4 GSCs were associated to the increased responsiveness to CAR T cell control (97). Confirmation papers are highly desirable to assess whether the same gene editing could potentially optimize the activity of CAR T lymphocytes characterized by antigenic specificity other than IL-13Rα2, in a CNS tumor context other than GSC.

***Epidermal growth factor receptor (EGFR/ErbB1/HER1)*** belongs to the tyrosine kinase receptor family, also including ErbB2, ErbB3, and ErbB4 (98). Overexpression of EGFR was described in many solid tumor types and associated with cancer cell resistance to chemotherapy (99, 100). In GBM, EGFR amplification is mostly associated with gene rearrangements associated to deletion of particular exons, or portions of exon, and leading to the generation of EGFR variants, that include deletion at N-terminal regional (EGFRvI), deletion at exons 14-15 (EGFRvII), deletion at exons 2–7 (EGFRvIII), deletion at exons 25–27 (EGFRvIV), deletion at exons 25–28 (EGFRvV). The EGFR-encoding gene is overexpressed in almost 50% of GBM patients, 25–64% of which present EGFRvIII (101, 102), making this oncogenic protein an optimal therapeutic target for CAR T cells (103). In a Phase I study (NCT02209376) conducted at the University of Pennsylvania and California (USA), 10 adult patients (45–76 years) affected by EGFRvIII-expressing recurrent GBM were treated with CAR. EGFRvIII T cells, incorporating 4-1BB as costimulatory domain (104). Patients received intravenous infusion of the product, without evidence of EGFR-directed “*off-tumor, on-target*” toxicity or systemic CRS. However, magnetic resonance imaging (MRI) show the lack of a marked tumor regression in the enrolled patients. Only one patient had residual stable disease for 18 months (104). This study underlined the difficulties of the clinical translation of EGFRvIII targeting, due to the intra-tumoral heterogeneity of the antigen expression, leading to clonal selection of the antigen-negative tumor cells. The antigen-escape phenomenon is mostly observed in highly heterogeneous tumors when a single antigen is targeted. This outcome seems to be related neither to the vector platform nor to the CAR design, since similar results have been observed in a Phase I pilot trial on 18 patients with GBM treated using autologous T cells genetically modified with a lentivirus carrying CAR.EGFRvIII/CD28.4.1bb (105). To optimize the CAR T cell therapy, a combinatory approach has been suggested. In particular, PD-1 checkpoint blockade achieved by either antibody blocking (106) or CRISPR-Cas9 (107–109) gene editing has been used in combination with CAR.EGFRvIII T-cells, both *in vitro* and *in vivo* models. To minimize “*on-target, off-tumor*” toxicity, Ravanpay et al. (110), proposed a second-generation CAR.EGFR designed using the scFv derived from the monoclonal antibody mAb806, specific for EGFR epitope present also in the EGFRvIII. Therefore, the construct binds to EGFR subjected to gene amplification, with no recognition of wild-type EGFR expressed on astrocytes (110). Recently, a Phase I study testing locoregional infusion of CAR.EGFR806-CAR T-cells has been opened to enrol paediatric patients with EGFR-positive recurrent/refractory CNS tumors (NCT03638167).

Choi et al. (111) proposed a CAR.EGFRvIII T-cells secreting EGFR-bispecific T-cell engager (BiTE) to circumvent antigen escape. In mouse models of GBM, they showed that CART.BiTE cells were able to secrete BiTEs locally in the brain tumor, redirecting non-specific bystander T cells against tumors which eliminated efficacy heterogeneous tumors more efficiently, compared to the use of a conventional CAR T cell approach (111). This group elegantly combined two therapeutic tools (CAR and BiTE), which are typically considered competitive technologies, which instead could be applied in a synergic manner.

***The human epidermal growth factor receptor 2 (HER2)*** has been described to be involved in cancer cell growth and metastasis, thus representing an attractive target for immunotherapy (112). The primary mechanism of HER2 activation in these cancers is the gene amplification leading to the overexpression of the HER2 on the cell membrane. The second mechanism recognized to drive the protein activation is related to the occurrence of activating mutations (113). HER2 is overexpressed in approximately 51% Wilms tumor, 44% bladder cancer, 26% pancreatic carcinoma and 25% breast carcinoma (114). ErbB2/HER2 mutation was also detected in 8–41% GBMs (115). Primary GBM stem cells are targeted by CAR.HER2 T, inducing regression of patient derived tumors in the animal model (116). In open-label Phase I dose-escalation trial, Ahmed et al. (117) used virus (CMV, Epstein–Barr Virus, and Adenovirus) specific T cells (VSTs), expressing a CAR.HER2.CD28.ζ construct to treat 16 HER2-positive relapsed/refractory GBMs. CAR-modified VSTs show persistence after the infusion for up to 12 months, using molecular DNA evaluation. Whereas the majority of treated patients relapsed early after the treatment, the overall response rate was of 50%, with the median OS of 11.1 months from the CAR VSTs infusion (117).

Lastly, Zhang et al., in a preclinical model targeting HER2, proposed a different platform to treat GBM patients, based on NK-92 cell line transduced with CAR.HER2.CD28.ζ construct. In the *in vitro* and *in vivo* models, they show a potent and specific lytic activity of the CAR NK-92 cell line toward GBM (118).

***Disialoganglioside GD2*** is a ganglioside (119) of unknown function, with limited expression in normal tissues and high expression in to neuroblastoma (120), melanoma (121), some sarcomas (122) and H3-K27M-mutated diffuse midline gliomas (DMG) (123). Intravenous anti-GD2 antibodies have already become the standard of care for high risk neuroblastoma patients (124, 125). Although it has been reported that a normal brain express low level of GD2 (126), in pre-clinical models (127) and clinical trials exploring the safety and efficacy of GD2-CAR T-cell whose scFv being derived from 14g2a monoclonal antibody (including our academic clinical trial NCT03373097), no cases of significant neurotoxicity were reported (128, 129). Mount et al. showed, that intravenously administered CAR.GD2 T-cells are able to cross the BBB and clear patient-derived H3K27M-mutated DMG tumors (123). Shum et al. showed that CAR.GD2 T cells also engineered to express IL-7 receptor (GD2-CAR.C7R) promotes CAR T cells persistence, proliferation and anti-tumor activity in an orthotopic GBM xenograft model (130). These encouraging studies have fastened the clinical translation of CAR.GD2 T cells in patients with brain tumors (NCT04099797, NCT04196413).

***B7-H3/CD276*** is a type I transmembrane protein belonging to the B7/CD28 superfamily, found in a wide variety of normal tissues and cells, including T and B cells, endothelial cells, resting fibroblasts, osteoblasts. Recently, several studies have proved the overexpression of CD276 on a variety of TME cells, as well as hematologic and solid tumors, including leukemia (131), prostate cancer (132), melanoma (133), neuroblastoma (134), osteosarcoma (135), and CNS tumors (136).

Many pediatric CNS tumors express B7-H3; in particular, high B7-H3 expression has been found in the following tumors: atypical teratoid/rhabdoid tumors **(**ATRT), ependymomas (all grades), MB, CNS embryonal tumors, choroid plexus tumors (CPTs), meningioma and craniopharyngiomas (136, 137).

In MB, WNT subtype expresses the highest B7-H3, while SHH subtype show the lowest one (136). However, low-grade gliomas and germ cell tumors show a negligible difference of B7-H3 expression respect to normal brain (136).

Majzner et al., recently reported that CAR.B7-H3 T cells incorporating 4-1BB as costimulatory domain, showed a potent efficacy in *in vivo* xenograft models, including MB (138). Loco-regional delivery of CAR.B7-H3 T cells also exerted significant activity over the systemic infusion for the treatment of CNS malignancies (139). In this study, CAR.B7-H3 T-cells used for intra-cerebroventricular or intra-tumoral administration, mediated a significant antitumor effects in the brain ATRT xenograft mouse model, with a reduced toxicity relative a significant diminished systemic levels of inflammatory cytokines as opposed to intravenously administered CAR T cells (139). The Seattle Children’s Hospital has started a clinical trial in 2019 based on CAR.B7-H3 T Cell based locoregional infusion in DIPG/DMG patients and recurrent/refractory paediatric CNS tumors (NCT04185038).

***CD70*** is a type II transmembrane protein, ligand for CD27. While activated T/B lymphocytes and a subset of mature dendritic cells express high level of CD70 expression, several hematologic and solid tumors, including GBM, constitutively overexpress CD70 (140–142). Indeed, CD70 expression is an independent predictor of poor OS for patients with low-grade gliomas (LGG) and GBM, correlating with chemokine-mediated immune modulation, tumor aggressiveness and immunosuppression *via* tumor-associated M2 macrophage recruitment/activation in GBM (142). Linchun et al., reported that CAR.CD70 T cells potently induce lytic activity against CD70+ gliomas, both *in vitro* and *in vivo* models (143). In order to increase the intra-tumoral T-cell migration, the same group integrated CXCR1 and CXCR2 chemokine receptors into the CD70-CAR approach (144). Moreover, to overcome the intra-patient variability, for which antigen heterogeneous expression on the tumor cells may result in a non-complete eradication of the tumor, and inter-patient variability, for which tumor cells from different patients may express different antigen patterns (145), bi-specific [CD70 and B7-H3 (146) or HER2 and IL-13Rα2 (147)], or even trivalent (targeting at the same time HER2, IL1-3Rα2 and EphA2) (148) CAR approaches have been developed, also in combination with DNA-demethylating agent azacytidine to diminish the occurrence of relapse after CAR T-cell administration in the animal xenograft models of Group 3 MB and ependymoma, associated to antigenic escape secondary to epigenetic silencing (149).

***CD-133*** also called Prominin-1, is a pentaspan membrane glycoprotein. Several human malignancies show OS inversely correlating with CD133 expression (150). Indeed, CD133^+^ cancer stem cells are known markers of chemo- and radio-resistance in GBM (151). Recently, Vora et al. provided evidences that, in a preclinical model, CAR.CD133 T cells, with CD28 costimulation, represent a therapeutic option to target self-renewing, chemo-radio resistant CD133^+^ brain tumor-initiating cells (151).

**Chondroitin sulfate proteoglycan 4 (CSPG4)** is a type I transmembrane protein with a central role in tumor progression and metastasis (152). Many types of solid tumors overexpress CSPG4, barely detectable in normal tissues (153–155). In preclinical models, CSPG4-directed CAR T-cell therapy efficiently inhibits GBM-derived neurospheres growth in both *in vitro*, as well as *in vivo* xenograft patient-derived GBM orthotopic mouse model (156). Clinical studies need to be conducted to study the safety and real therapeutic activity of the proposed innovative CAR approaches.

## Immunomodulation

Approaches to optimize endogenous T-cell immune responses, including Immune Checkpoint Inibitors (ICIs) (157, 158), oncolytic viruses (159), and tumor neoantigen vaccines (160) have shown evidence of bioactivity against brain tumors, also in clinical trials (**Figure 1** and **Table 1**).

### Immune Checkpoint Inhibitors (ICIs)

Although immune checkpoint inhibition using antibodies against CTLA-4, PD-1 or PD-L1 has shown considerable tumor regression in several solid tumors, CNS tumors remain refractory to these treatments (161). ICI administration in melanoma patients with CNS metastases has shown a durable anti‐tumor response (162). This positive outcome could be explained considering that melanoma has a high tumor mutational burden (TMB) and that TMB has been clearly correlated to ICI response in treated patients. In line with these observations, also a subset of GBMs characterized by high TMB, show a durable remission after ICIs (157, 158). However, the majority of brain tumors are characterized by low TMB, this last explaining a low overall responsiveness to ICIs (163). It has been suggested that radiation and chemotherapy therapies in newly diagnosed CNS tumors could increase the TMB and therefore, favor the response to ICIs (164), as also proved in an animal model (165). However, recent phase III trials (CheckMate-143 and CheckMate-498) failed to prove the OS improvement in GBM patients receiving PD-1 monotherapy (166). Nevertheless, these studies remain important as they confirm that CNS tumors are accessible to the immune system, and were associated with some clinical benefit, including the induction of the so-called “abscopal effect”. This phenomenon is linked to the recognition and elimination of tumor cells outside the irradiation field that are induced by the immune system when ICI are administered (167). Thus, further clinical studies are required to define the optimal application of ICI in GBM patients, considering the peculiarity of the target tumors. In particular, several reasons could explain this unsatisfactory outcome, including the lack of prominent T cell infiltrates especially observed in to mismatch repair-deficient gliomas (168), the paucity of DC in the brain TME playing a key role in the recruitment of T cells (57, 169–172), and the reduced ability of monoclonal antibodies to cross effectively the BBB (173, 174). Several attempts are under evaluation to circumvent this latter aspect, for example, by the delivery of ICI by nanoparticles (175), to deliver small interfering RNAs (siRNAs) targeting EGFR and PD-L1 (176). In a combinatory point of view, it has been suggested that brain delivery of nanoparticle can be potentiated by low-dose radiation that altering the tumor structure allows for the enhanced nanoparticles delivery in tumor-associated macrophage-dependent fashion (177). In regard to the issue of a reduced contribution of DC in the brain TME, the role of APC in controlling response after ICI is strongly suggested by several evidences. Indeed, as in the model of skin carcinoma in which it was proved that T cell response to ICI is associated to the expansion of T cells that have entered de-novo the tumor instead of the re-activation of pre-existing highly-exhausted TIL (178, 179). It could also be plausible that the clinical response to ICI may be associated to the newly recruitment of T cells to the tumor site that are induced by the signal generated from activated APCs. Further investigations to study the clinical efficacy of combinatory therapy between CARs and ICI are ongoing, including a clinical trial in GBM patients (NCT03726515) that will evaluate the combination of CAR.EGFRvIII T cells in association with pembrolizumab (PD-1 inhibitor).

### Oncolytic Virus (OV) Approaches

OV with their ability to selectively replicate in tumor cells, has been described as potent antitumor approach (3). GBM was shown to be responsive to OVs in preclinical models and clinical trial (180–182). To overcome the viral exclusion exerted by the BBB, the OVs can be intratumorally injected, or the approach can be based on parvovirus that is known to have good CNS penetration (183). Pivotal trials have established the safety of OVs in GBM, but their efficacy has been modest to date (184). Innovative approaches are focusing on targeted OVs and combinatory therapy. GBM stem cells utilize autophagy modulation as main resistance mechanism to OVs (185). Indeed, glioma oncolysis is significantly reduced when Beclin-I (autophagy inducer) is downregulated in tumor cells, and tamoxifen drug, upon activation of apoptosis in OV infected cells *via* BAX/PUMA pathway, is able to re-establish the tumor sensitivity to oncolytic infection (185). Oncolytic HSV-1 (oHSV) G207 has been the most comprehensively investigated virus in human brain tumors (186–190). Phase I studies in adult patients with recurrent GBM provided data of safety for intratumoral injections of single (186) or double doses of G207 dosages (187) also combined with a single 5 Gy dose of focal radiation (188). Based on the safety observed in the trials on adult patients, a paediatric clinical trial (3–18 years old) in recurrent/progressive supratentorial malignant brain tumors has been opened (189).

The oncolytic adenovirus DNX-2401 (Delta-24-RGD) has been infused by single intratumoral injection in patients with GBM. Amongst the 37 treated patients, 20% experienced long term survival (>3 years from treatment), with three patients showing a significant tumor reduction (≥95%). These relevant clinical results were also corroborated by the observation of DNX-2401 replication and spreading within the tumor (191). The same oncolytic adenovirus DNX-2401 has been also used to treat pediatric subjects with DIPG, showing improvement in OS and quality of life (192). Lastly, Rat H-1 parvovirus (H-1PV) in recurrent glioblastoma patients has shown the ability to trigger specific T-cell responses (193).

### Vaccine-Based Approaches

The immune system can eradicate malignant cells *via* recognition of TAAs. Ideal TAAs have an expression restricted to tumor cells, being negligible in surrounding healthy tissue. In CNS tumors, tumor antigens can be categorized into three main classes; (i) antigens with an aberrant expression; (ii) mutated oncogenes and (iii) neo-antigens. One valid example is represented by Rindopepimut, an EGFRvIII-targeted vaccine, which tested in a clinical trial, failed to show survival improvement (194). Several other vaccines are in development with several promising candidates (195).

HSPPC-96, a vaccine based on heat-shock tumor peptides, has been applied to recurrent GBM in a phase II trial, showing 6-month survival rate of 90% (196). A different vaccination approach has been developed for AFTV, which is based on formalin-fixed tumor sample fragments, infused intradermal in GBM patients. This approach allows to reach a 3-year survival rate in 38% of the treated patients (197).

Autologous DCs have been also considered in the vaccination prospective. As previously underlined, CNS TME is characterized by a marked absence of infiltrating APC. For this reason, adoptive transfer of T-cells alone could not be substantially beneficial for patients with CNS tumors. Several approaches aimed at overcoming this CNS tumor limitation have been described. Current evidence deriving from phase I and II trials suggests that DC vaccination may activate an immune response against CNS tumors (198–202). Although more than 500 GBM patients have been treated with DC vaccination and clinical results have been encouraging, there is not robust evidence of clinical efficacy, because of either the non-controlled design of the studies or the low patient numbers. One attempt has been carried out by German clinical centers participating to the randomized controlled phase II trial GlioVax (mature DCs loaded with tumor lysate) (203). This trial has been designed to enroll 136 GBM patients into two cohorts (1): radio/temozolomide “gold standard therapy” and (2) DC vaccination plus standard therapy. Data coming from this trial will clarify the impact of DC vaccination on patient outcome.

## Combination Approaches

It is increasingly evident to researchers that a combination of different therapeutic approaches is needed to efficiently treat patients with high immunosuppressive brain tumors. Saha et al., showed that the triple combination of an OV expressing IL-12 with two immune checkpoint inhibitors targeting PD1 and CTLA-4, can eradicate glioma in two mouse models (204), reducing Tregs, increasing CD8+ T cells and inducing M1-like polarization in TAMs. Moreover, in a *in vivo* model of Glioma, Tang et al., proposed a complex therapy based on a combinatory approach of an adoptive cell transfer of tumor-specific CD8+ cells (to increase adoptive T cell targeting of the tumor), rapamycin (to enhance antigen presentation by DCs), celecoxib (to modulate the TME inflammation and immunosuppression), and intratumoral injection of IL15Rα-IL15-armed oncolytic poxviruses (to boost T cell activation) (205). In tumor-bearing mice, the combinatory approach was safe and able to promote longer survival of tumor-specific cytotoxic T cells, in respect to the administration of only CD8+ T cells (205). Pivotal clinical trials need to be designed and carried out to corroborate the safety and efficacy of the described combinatory approach.

## Conclusions

The recent advances in immunotherapies, coupled with the essential insights in the understanding of neuro-immunology, are creating innovative opportunities to treat CNS cancer. To achieve durable antitumor effect, it is likely that combinatorial regimens will be required and based on 1) increase of CNS delivery; 2) “multi-valent” tumor targeting; 3) targeting both tumor cells and the CNS TME. The road to identify an effective cure for brain tumors still appears long and filled with pitfalls, but the deep knowledge of these tumors and their microenvironment will lead in the near future to increasingly personalized treatment, possibly changing the natural history of these diseases.

## Author Contributions

CQ rearranged the final version. BDA coordinated the coauthor’s contribution and rearranged the final version. All authors contributed to the article and approved the submitted version.

## Funding

We would like to thank all agencies supporting our work on immunotherapy, in particular the Italian Health Ministry [for GR-2013-02359212, (CQ), GR-2016-02364546- (BDA), CAR T RCR-2019-23669115]; AIRC; Ricerca Corrente (CQ, BDA); All the charities that support our research and clinical translation, including “Raffaele Passarelli” Onlus, “Il laboratorio di Chiara,” “Fondazione Heal,” “IRENE” Onlus, “Oscar’s Angels Italia,” and “Martina e la sua luna.”

## Conflict of Interest

The authors declare that the research was conducted in the absence of any commercial or financial relationships that could be construed as a potential conflict of interest.
